# Secondary Glioblastoma Multiforme in a Child with Disseminated Juvenile Pilocytic Astrocytoma

**DOI:** 10.1155/2012/290905

**Published:** 2012-11-19

**Authors:** C. S. Amene, L. A. Yeh-Nayre, J. R. Crawford

**Affiliations:** ^1^The Departments of Neurosurgery, San Diego and Rady Children's Hospital, University of California, 3020 Children's Way San Diego, San Diego, CA 92123, USA; ^2^The Departments of Pediatrics, San Diego and Rady Children's Hospital, University of California, 3020 Children's Way San Diego, San Diego, CA 92123, USA; ^3^The Departments of Neurosciences, San Diego and Rady Children's Hospital, University of California, 3020 Children's Way San Diego, San Diego, CA 92123, USA; ^4^Division of Child Neurology, Rady Children's Hospital San Diego, University of California San Diego, 8010 Frost Street Suite 400, 3020 Children's Way San Diego, San Diego, CA 92123, USA

## Abstract

Secondary glioblastoma multiforme (sGBM) can occur after a long latency period following radiation treatment of various diseases including brain tumors, leukemia, and more benign disorders like tinea capitis. Outcomes of radiation-induced sGBM remain poor in both children and adults. We report a case of a 16-year-old girl with a history of disseminated juvenile pilocytic astrocytoma treated with chemotherapy and craniospinal radiation 9 years prior who developed sGBM in the absence of a tumor predisposition syndrome. She presented with a several-week history of headaches and no acute findings on computed tomography compared to baseline neuroimaging 3 months prior. Repeat computed tomography performed just 3 weeks later for worsening headaches revealed a new large posterior fossa tumor where pathology confirmed the diagnosis of sGBM. In spite of maximal surgical resection, reirradiation, and adjuvant chemotherapy, she died 1 year postdiagnosis. Our case highlights the potential late effects of high-dose cranial radiation, how symptomatology may precede neuroimaging findings, and the rapid formation of sGBM that mirrors that of *de novo* Glioblastoma Multiforme.

## 1. Introduction

Radiation remains the mainstay of therapy in a wide variety of both primary and metastatic central nervous system (CNS) tumors in both children and adults. Its potential long-term deleterious effects are well documented and include radiation necrosis, neurocognitive sequelae, vasculopathy, and development of secondary malignancy, most notably secondary glioblastoma multiforme (sGBM) [1]. Newer radiation techniques of intensity modulated radiation therapy and stereotactic radiosurgery have not decreased the likelihood of sGBM [2]. Radiation-induced malignancies were initially described by Cahan et al. in 1948 to include the following criteria: (1) tumors arising from a prior radiation field, (2) there must be a latency period, usually years, between the radiation therapy and onset of secondary tumor, (3) there must be histopathologic difference between the primary disease treated and the secondary tumor and, (4) the patient must not have any carcinogenic disease (e.g., Tuberous sclerosis, Li Fraumeni syndrome, and Neurofibromatosis) [3]. The first reported cases of radiation-induced CNS tumors were in the 1950s and were noted to be mostly fibrosarcomas and meningiomas. Cahan's definition has since been extrapolated to include radiation-induced sGBM.

The actual risk of sGBM and other radiation-induced malignancies is not well known, however in a study of pediatric patients irradiated for tinea capitis, it was found to be approximately 2.6-fold increased compared to nonirradiated historical controls [4]. Based on the limited number of case reports and series of sGBM described in the literature, there is a higher incidence in younger patients (60–75% of cases), radiation volume was more important than total radiation dose, sGBM often occurred in the suprasellar area and the cerebellum (in direct contradiction to *de novo* GBM), and the most common etiologies were radiation therapy for brain tumors and leukemia [5–8]. A recent study by Paulino et al. reviewing almost 100 cases of radiation-induced sGBM revealed a median survival in of 9 months with a 0% 5-year-survival rate [5]. We report a the case of sGBM in a young girl that developed 9 years after initial radiation therapy for disseminated suprasellar juvenile pilocytic astrocytoma (JPA) in the absence of a history of an underlying tumor predisposition syndrome. Our case of sGBM illustrates: (1) the rapid growth velocity and insidious onset of sGBM, (2) how symptomatology can precede neuroimaging findings, and (3) the refractory nature of sGBM to re-irradiation.

## 2. Case Report

**Figure 1:**
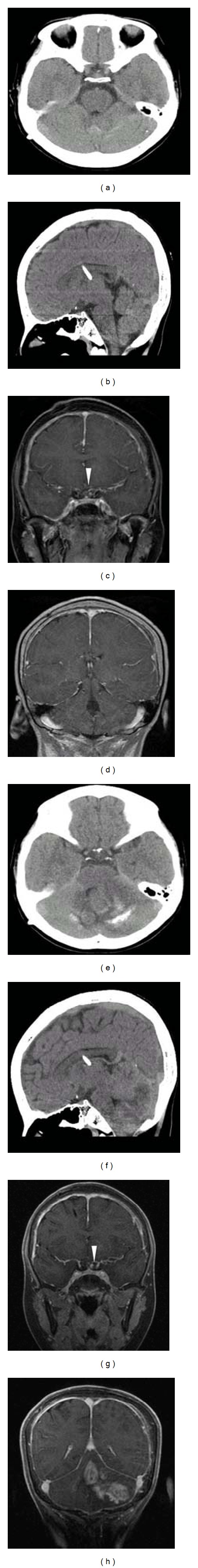
Neuroimaging findings of sGBM. Axial noncontrast CT shows stable changes of mineralization of the posterior fossa without hydrocephalus 3 weeks before tumor recurrence (a) and (b). Postgadolinium coronal MRI (c) shows stable suprasellar disease at baseline (arrow) and no evidence of cerebellar mass 3 months prior to relapse (d). Axial and coronal noncontrast CT at relapse 3 weeks following initial CT shows a large cerebellar tumor with extensive edema and compression of the 4th ventricle (e) and (f). Postgadolinium MRI confirms the CT findings with a stable suprasellar mass (g) compared to baseline and a new large posterior fossa cerebellar tumor (h).

**Figure 2:**
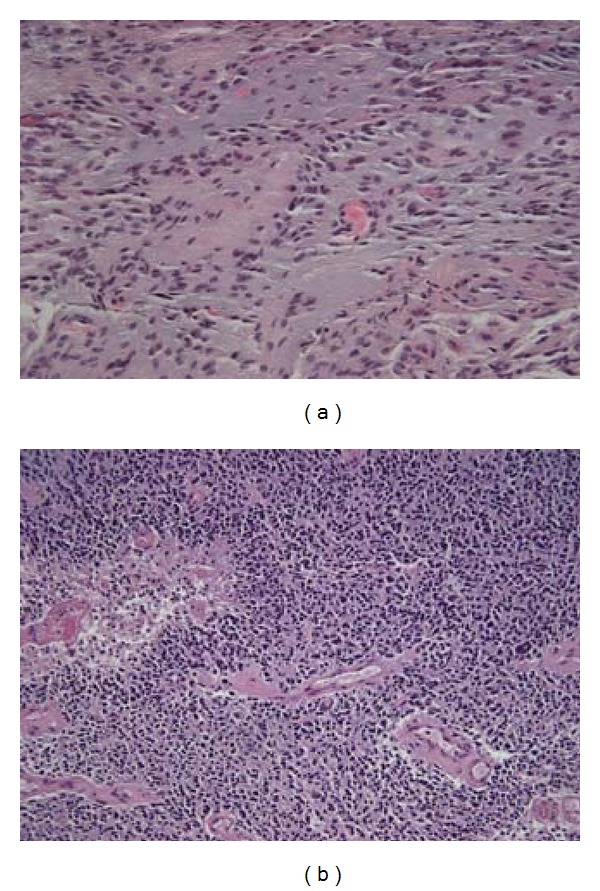
Histopathology of Primary JPA and sGBM. Tumor at original diagnosis reveals a hypercellular biphasic pattern of solid and cystic neoplasm with bland nuclei, and microvascular proliferation consistent with a diagnosis of low-grade glioma (a) (40x magnification, hematoxylin-eosin stained) Posterior fossa tumor 9 years after initial diagnosis reveals nuclear atypia with numerous mitosis, vascular proliferation, and pseudopalisading necrosis consistent with a diagnosis of glioblastoma multiforme (b) (20x magnification, hematoxylin-eosin stained).

A 16-year-old girl with history of suprasellar disseminated juvenile pilocytic astrocytoma treated 9 years prior with chemotherapy and craniospinal radiation as previously reported [9], presented with a 1 day history of headaches and vomiting. She had been seen 2 weeks prior in neuro-oncology clinic where her examination revealed baseline paraplegia with hyporeflexia and neurogenic bowel/bladder from her previous spinal cord involvement. Her computed tomography (CT) scan revealed stable mineralization of the posterior fossa with no changes in the suprasellar region or posterior fossa and no interval hydrocephalus (Figures 1(a) and 1(b)), similar to her last magnetic resonance imaging (MRI) just 3 months prior (Figures 1(c) and 1(d)). Three weeks later when her headaches did not resolve a repeat CT scan was performed that showed a new large cerebellar mass with edema and compression of the 4th ventricle (Figures 1(e) and 1(f)). MRI confirmed the findings of a posterior fossa tumor on CT (Figure 1(h)) without changes in the size of the stable suprasellar JPA (Figure 1(g)). She underwent gross total resection where histopathologic analysis revealed an sGBM, characterized by pseudopalisading necrosis, vascular proliferation, and high mitotic index that was markedly distinct from her initial biopsy-proven diagnosis of JPA 9 years prior (Figure 2). Her pathology at initial diagnosis revealed a classic JPA architecture with a biphasic pattern of solid and cystic glial neoplasm with Rosenthal fibers, and Eosinophilic Granular Bodies (Figure 2(a)). Postoperatively, she underwent treatment with a combination of therapies including reirradiation, temozolamide, bevacizumab, and everolimus. Unfortunately, she died 1 year postdiagnosis of sGBM and 10 years post her initial diagnosis of disseminated JPA.

## 3. Discussion

The higher frequency of radiation-induced malignancies seen in children may be due to an increased inherent radiosensitivity and the long latency period between treatment and development of the secondary tumor [10]. A conservative approach is generally taken in managing cases of pediatric low-grade tumors since JPA is a WHO grade I tumor and is curable in many cases by complete surgical resection. In cases where complete surgical resection is not possible, or in cases of disseminated disease, radiation therapy and chemotherapy are often utilized [11]. Disseminated JPA is very rare occurring in about 4-5% of all cases of JPA, and chemotherapy and radiation therapy are common treatment modalities that have been used to stabilize disease progression [11–13]. As was the case with our patient, given her extensive disease at initial presentation, and her lack of chemotherapy responsiveness, craniospinal radiation provided significant disease control of her disseminated JPA for 9 years prior to the diagnosis of sGBM [8].

Studies analyzing the molecular basis of radiation-induced GBM have shown no difference between sGBM and *de novo* GBMs [14, 15]. However, there may be more homogeneity in gene expression in sGBMs compared to de novo GBMs [15]. Another difference reported is their anatomical distribution. Although sGBM occurs more often in the temporal and frontal lobe like their *de novo* counterparts, sGBM may also occur in suprasellar and infratentorial regions, a very rare occurrence in primary GBMs. Other reported differences include higher incidence of multifocality in sGBM and more aggressive clinical behavior [7].

The relatively early detection of our patient's sGBM in light of the normal CT neuroimaging study 3 weeks prior, did not affect overall survival. This is consistent with findings by Paulino et al., who reported no differences in overall survival between patients who underwent maximal surgical resection of sGBM or who received adjuvant chemotherapy [5]. The use of re-irradiation therapy to treat sGBM is controversial as there is concern about potential acute and subacute CNS toxicities such as refractory cerebral edema and intra-tumoral hemorrhage. However, reirradiation has been shown to improve overall survival in a small series of adult patients with sGBM when compared to those patients who were not reirradiated and is the subject of ongoing clinical trials in both adults and children [5].

One of the most unique aspects of our case report is the rapid onset of tumor formation in the setting of a normal noncontrast head CT just 3 weeks prior. It is very likely that if an MRI were performed at the time of her initial headache, it may have shown some subtle T2 or post gadolinium T1 changes. However, her MRI 3 months prior showed no such abnormalities. Our patient's symptomatology preceded the CT neuroimaging findings by 3 weeks despite an unchanged neurological exam. This highlights the importance of assessing symptomatology as part of routine screening for the presence of secondary malignancies. It is our practice to screen all children who have received cranial radiation yearly for the presence of secondary malignancies and vascular malformations. However this case illustrates that symptoms such as headache, nausea, or vomiting, even in the setting of an unchanged neurological exam may warrant early MRI surveillance imaging.

## 4. Conclusion

In children who have received cranial radiation, sGBM remains a considerable challenge with regards to both diagnosis and treatment. The onset of sGBM may occur over a period of weeks and symptomatology may precede neuroimaging findings.
